# Innate colour preference in zebrafish (Danio rerio)

**DOI:** 10.1016/j.mex.2023.102342

**Published:** 2023-08-23

**Authors:** Ethan V. Hagen, Yanbo Zhang, Trevor J. Hamilton

**Affiliations:** a Department of Psychiatry, 5-065 Katz Group Centre for Pharmacy and Health Research, University of Alberta, Edmonton, AB T6G 2E1, Canada; b Department of Psychology, MacEwan University, 10700 104 Ave NW, Edmonton, AB T5J 4S2, Canada; c Neuroscience and Mental Health Institute, University of Alberta, Edmonton, AB T6G 2E1, Canada

**Keywords:** Behavioural neuroscience, Conditioned preference, Sex difference, Plus maze, Fish behaviour, Behavioural testing, Colour preference testing.

## Abstract

Innate (natural) colour preference in animals is used for a variety of behavioural neuroscience purposes in many animal models. In zebrafish, colour preference is often used in combination with place preference testing and some memory tests. However, baseline colour preference seems to differ in the few studies examining this innate behaviour. This necessitates a protocol for reliable colour preference testing to establish preferences prior to using more complex behavioural paradigms. This procedure involves an aquatic plus maze with a central neutral zone and 4 coloured zones: red, green, yellow, blue. Adult zebrafish spent significantly more time in the blue zone compared to the red and yellow zones. There were no sex differences in colour preference. This procedure is a rapid, affordable, straightforward, and effective method to establish baseline colour preference.

## Graphical abstract

**Figure:**
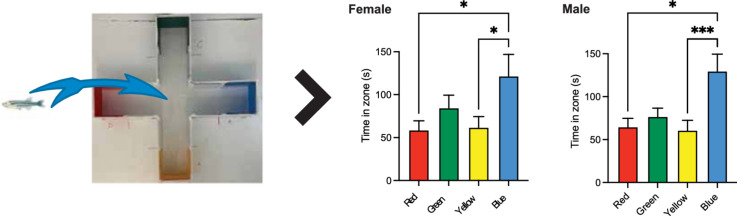

Specifications table

**Table:** 

Subject area:	Psychology
More specific subject area:	Zebrafish Behavioural Neuroscience
Method name:	Colour preference testing.
Name of your protocol:	Plus maze colour preference test
Reagents/tools:	– Plus maze – Red, green, yellow, blue, white corrugated plastic cut to size of maze – Motion-tracking software – Data analysis software
Experimental design:	Fish are placed into a plastic 4-way maze lined with 4 colour options on arms of the maze. Motion tracking software records the time spent in each arm of the maze and zebrafish preference is determined.
Trial registration:	*N/A*
Ethics:	*Zebrafish experiments completed with the ARRIVE guidelines and were carried out in accordance with the Canadian Council on Animal Care Canadian guidelines for experimental animals and was approved by the Grant MacEwan Animal Research Ethics Board (101853).*
Value of the Protocol:	– **Materials:** Acrylic plus maze (∼CAD$150; made in-house), 24” X 48” sheets of coloured corrugated plastics (Coroplast, QC, Canada; $50/ per sheet), seedling heat mats (iPower; 20$ per mat),cal-SPOT 401 photometer (The Cooke Corporation, MI, USA; ∼$2000). – **Computer system and software:** EthoVision motion tracking software system including camera and computer (Noldus information technology, VA, USA;∼$15000), Graphpad Prism (CA, USA; ∼$1000).

## Description of protocol

Zebrafish have become a useful and popular research model in behavioural neuroscience, pharmacology, neuropsychiatric, and toxicology studies [4,6]. Many studies involve coloured stimuli with varying complexity, saliency, and relevance to the experimental protocol being tested [8]. Some studies have examined colour preferences in zebrafish [1,^2^,^7^,^9^,10] but the results vary from these experiments, necessitating further examination of innate colour preference in zebrafish.

This apparatus uses a plus maze format allowing for 4 different coloured arms and a neutral center zone. The colours used here are red, yellow, blue, and green. Each arm has a length of 21 cms with a width of 11 cms. To avoid any directional preference the location of the colours were switched every water change (every ∼4 fish). An overhead camera connected to a motion tracking software system is used to quantify the time they spend in the coloured zones. This protocol describes use with zebrafish, however, any fish species could be used.

*Establishing a colour preference test using easily obtainable and affordable materials is important in building a repeatable model that can be used worldwide.*

1. Remove fish from the housing tank/system and place into smaller housing tanks (10-15 per tank) to be transferred to the testing room (Fig. 1A).

**Fig. 1:**
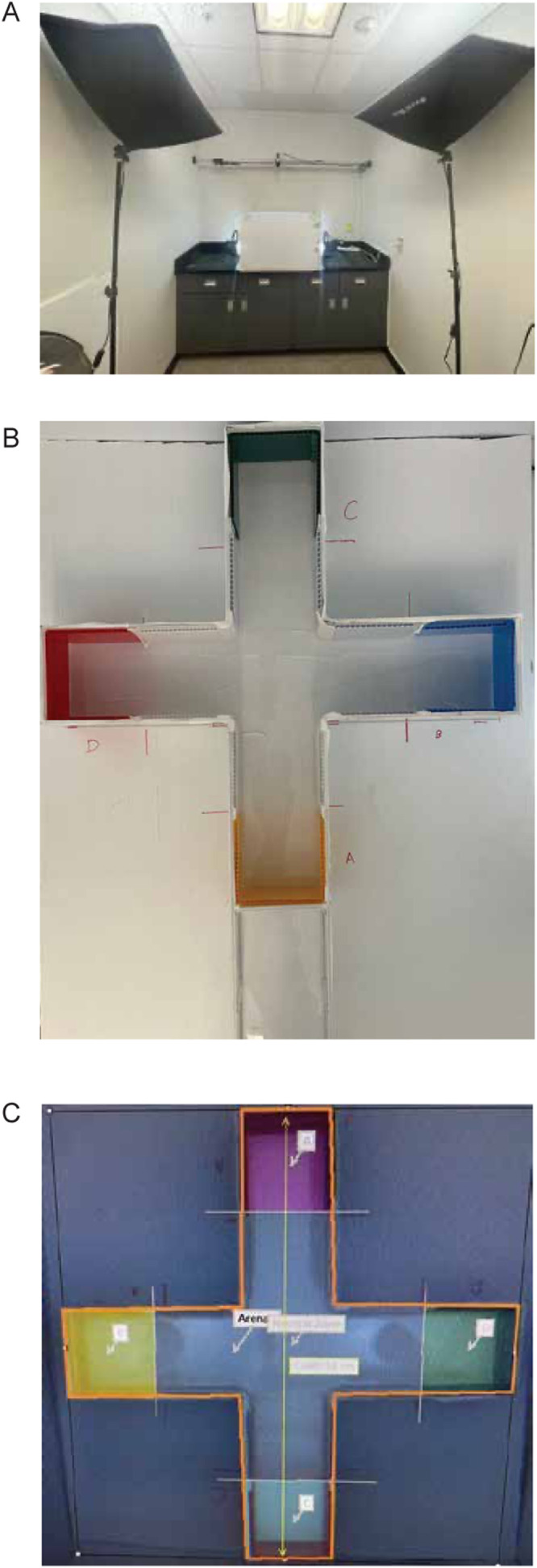
Colour preference testing set-up. (A) Image of fish testing room. Camera is placed above the arena which is enclosed within white corrugated plastic. (B) Image of plus arena with coloured sheets (red, blue, yellow, red) placed on each of the arms. Each arm is 11 cm wide and 21 cm long. The height of the walls are 11 cm. (C) Image from EthoVision demonstrating the zones used for data analysis.
2. Allow 20 min of acclimation time in the testing room. Fish tanks, beakers, and the plus maze are placed on heat mats to maintain the temperature of the water.
3. Fill plus maze to a height of 5cm with habitat water.
4. Place plastic coloured sheets at the ends of each part of the plus maze (4 colours) (Fig. 1B). We place the plastic sheets within the water to avoid potential differences in reflectivity with placement outside the plus maze.
5. Set up motion-tracking software system (Fig. 1C). We suggest the differencing detection method in EthoVision. Ensure the testing arena is visually separated from other stimuli within the testing room. We use white corrugated plastic to shield the plus maze (Fig. 1A). Test luminance of the room (we use a CalSpot photometer and ∼32 cd/m^3^) and calibrate if needed.
6. Place a single fish into the center of the plus maze (neutral area).
7. Record and track the fish for a duration of 10 min.
8. Remove fish from arena and place into a small beaker containing habitat water to determine sex of fish. After allowing the fish to acclimate in the beaker (this allows full colour to return to the fish) sexing is done primarily via two criteria. Firstly, investigate the colour of the fish: males have a yellow anal fin whereas females have a clear anal fin. Female fish also have a more prominent silver belly compared to males [9,10]. Secondly, investigate the shape of the fish: female zebrafish will typically have a larger abdomen region compared to the slenderer male body shape (Fig. 2). Following sexing place fish into a separate tank with other tested fish.

**Fig. 2:**
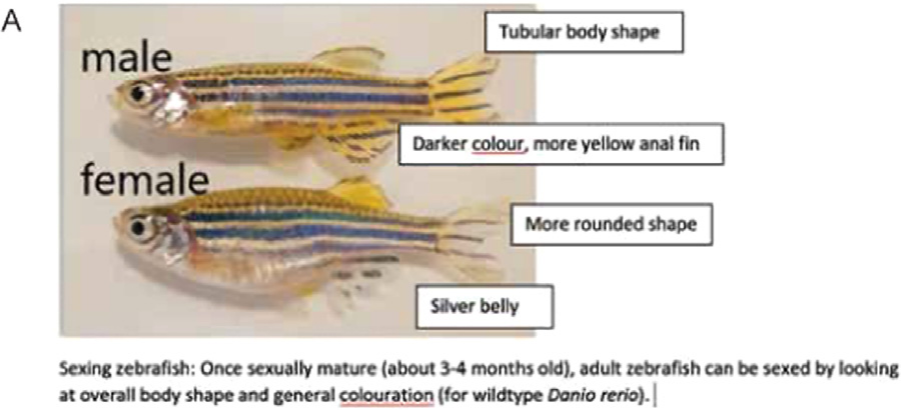
Sex determination in zebrafish (*Danio rerio*). (A) Image of male and female zebrafish and characteristics used to determine sex. Males have a longer and thinner tubular body shape and are darker in colour with a more yellow anal fin. Females have a rounder body shape and a silver belly.
9. Change arena water minimally every (3-5 fish) and randomly rotate the colours in the 4 quadrants to prevent directional preference. Using the same water in the testing apparatus for up to 5 fish does not produce behavioural differences on anxiety-like behaviour [5]. Check and maintain the water temperature in between trials if water changes are not performed after every fish.
10. Analyze data and quantify time spent in each zone of the plus maze. Test for normality using D'Agostino & Pearson test. Depending on the normality of the data, compare with a one-way ANOVA (parametric) or Kruskal-Wallis test (non-parametric) and the appropriate post-hoc test.
11. Note that we do not feed fish until after behavioural testing because this can alter behavioural responses [3].

## Protocol Validation

**Fig. 3:**
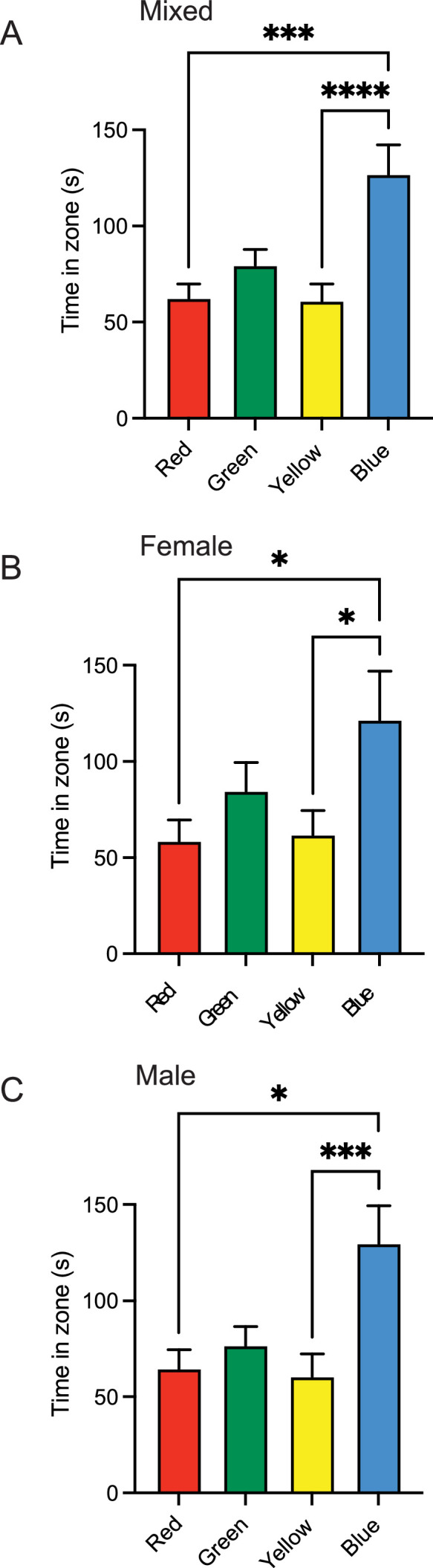
Innate colour preference in zebrafish (*Danio rerio*). (A) Time all fish spent in each coloured zone during the 10 min trial. (B) Time that the female fish spent in each coloured zone. (C) Time that the male fish spent within each coloured zone. Data was analyzed with a Kruskal-wallis test and Dunn's multiple comparison post-hoc test. *P < 0.05; *P < 0.01; ***P < 0.001; P < 0.0001.

To provide support for this method a test on the innate colour preference of zebrafish was conducted. In this study 55 adult zebrafish (*Danio rerio;* 35 males, 20 females) were tested using the above-mentioned methods. Fish were acclimated to the testing room for a duration of 20 min. Following this one fish at a time was placed into the colour preference plus maze for a duration of 10 min. After testing fish are set aside in a beaker and sexed based on body shape and anal fin colour (set aside for ∼10 min). Following sexing the fish is placed into a new habitat tank. Once all fish are tested the fish are fed and returned to their habitat room. All data was then quantified in the EthoVision program and exported to an excel file. Data from this excel file was then used in GraphPad Prism to perform statistical analysis, specifically a one-way ANOVA for parametric data or a Kruskal-Wallis test for non-parametric data. Using these methods, we were observed a statistically significant preference for blue zone compared to red and yellow zones for all fish (H(3)=26.13; P<0.0001). When investigating colour preference in each sex we observed the same blue preference in both females (H(3)=12.01; P=0.0074) and in the males (H(3)=16.1; P=0.0011) (Fig. 3).

## CRediT authorship contribution statement

**Ethan V. Hagen:** Conceptualization, Methodology, Data curation, Formal analysis, Methodology. **Yanbo Zhang:** Conceptualization, Supervision, Writing – review & editing. **Trevor J. Hamilton:** Supervision, Conceptualization, Funding acquisition, Writing – review & editing.

## Declaration of Competing Interest

The authors declare that they have no known competing financial interests or personal relationships that could have appeared to influence the work reported in this paper.

## Data Availability

Data will be made available on request.
